# Squawks in interstitial lung disease prevalence and causes in a cohort of one thousand patients

**DOI:** 10.1097/MD.0000000000016419

**Published:** 2019-07-19

**Authors:** Carlos A.C. Pereira, Maria R. Soares, Rafaela Boaventura, Marina D.C. Castro, Paula S. Gomes, Andrea Gimenez, Cesar Fukuda, Milena Cerezoli, Israel Missrie

**Affiliations:** a Interstitial Lung Diseases Program, Pulmonology Service; b Radiology Service, São Paulo Federal University, São Paulo, Brazil.

**Keywords:** bronchiolitis, hypersensitivity pneumonitis, interstitial lung diseases, lung sounds

## Abstract

Squawks are lung adventitious sounds with a mix of both musical and nonmusical components heard during the inspiratory phase. Small series have described squawks in interstitial lung diseases. Hypersensitivity pneumonitis and other diseases involving small airways can result in squawks, but new interstitial lung diseases (ILDs) involving peripheral airways are being described. A retrospective analysis was performed on 1000 consecutive patients from a database of ILD of a tertiary referral center. Squawks were recorded in 49 cases (4.9%), hypersensitivity pneumonitis (23 cases), connective tissue disease (7), microaspiration (4), pleuroparenchymal fibroelastosis (4), fibrosing cryptogenic organizing pneumonia (, 3), familial ILD (2), sarcoidosis (2), idiopathic pulmonary fibrosis (IPF; 1), bronchiolitis (2), and nonspecific interstitial pneumonia (1). One patient had a final diagnosis of IPF. There was a significant association between mosaic pattern and squawks: 20 cases with squawks (40.8%) had mosaic pattern compared with 140 (14.7%) cases without squawks (*x*^2^ = 23.6, *P* < .001).

Findings indicative of fibrosis were described on high-resolution chest tomography (HRCT) in 715 cases (71.5%). Squawks were more common in patients with findings indicative of fibrosis on HRCT: 45 of 715 (6.3%) compared with 4 of 285 (1.4%) of those without findings indicative of fibrosis (*x*^2^ = 10.46, *P* = .001).

In conclusion, squawks are an uncommon finding on physical examination in patients with ILD, but when present suggest fibrosing ILD associated with bronchiolar involvement. However, squawks are rare in IPF.

## 1 Introduction

Interstitial lung diseases (ILDs) are a diverse group of conditions that affect the lung parenchyma, with varying degrees of inflammation and fibrosis. In Brazil and other countries, fibrotic hypersensitivity pneumonitis (HP) is common, and its differentiation from idiopathic pulmonary fibrosis (IPF) is critical.^[1,2]^

Chest auscultation remains an important method of clinical assessment in patients with respiratory disorders, providing immediate and reliable information to clinicians.^[3]^

Squawks are adventitious sounds with a mix of both musical and nonmusical components heard during the inspiratory phase.^[3,4]^ Squawks are also called short wheezes or end-inspiratory wheezes as the sounds’ characteristics are similar to a wheeze but with a shorter duration.^[3,5]^ Other names used to describe this sound are squeaks and chirping rales.^[6]^

Geddes et al^[7]^ reported squeaks in 6 patients with bronchiolitis obliterans associated with rheumatoid disease. Earis et al^[8]^ assessed inspiratory squawks in 14 people with diffuse ILD, 9 of whom had HP. In 20 patients characterized as suffering from IPF, the authors found no cases with squawks.

These data suggest that squawks can be helpful in differentiating between HP and IPF, but new ILDs involving peripheral airways are being described, so the differential diagnosis could be larger in scope.^[9–11]^

The objective of present study was to assess the association of squawks with different ILDs and also with clinical and radiological characteristics in a group of patients referred to a tertiary ILD center.

## 2 Methods

A retrospective observational analysis was performed on 1000 consecutive patients from a database of ILD from Federal University of São Paulo.

A standardized protocol for investigation of ILD was used in all cases: complete clinical history and physical examination, high-resolution chest tomography (HRCT), serologic tests for autoimmune diseases, lung function tests, and exercise test (6-minute walking test or 4-minute step test).^[12,13]^ Patients included were cases evaluated at the first appointment by an ILD postgraduate (54%) or by the first author of present article (46%). All patients were auscultated during the first evaluation. Bilateral auscultation of the lungs was performed from the upper regions to the basal areas.

All research fellows were trained in detecting abnormal lung sounds, including wheezes, Velcro crackles and squawks, by using an audio CD on lung sounds.^[5]^ Squawks were characterized as a short duration squeaky sound listened at the final of inspiration, similar to the sound issued by birds.

### 2.1 Exclusion criteria

Patients with undefined diagnoses, those without chest HRCT available for evaluation and patients with sarcoidosis with the absence of lung parenchymal abnormalities on chest HRCT, were excluded. In our center, approximately 10% of ILD remain with undefined diagnoses. A patient with IPF and squawks with concomitant infectious pneumonia at initial evaluation was excluded from the final analysis.

### 2.2 Inclusion criteria

Patients evaluated for the first time between January 2010 and August 2018 with ILD. All diagnoses were established following the standard criteria.^[1,14–16]^ More complex cases were diagnosed in a multidisciplinary discussion that includes the presence of chest radiologists and specialized pathologists.

The diagnosis of HP was according to the criteria suggested by Salisbury et al^[17]^ for likely HP applied retrospectively.

Criteria for the diagnosis of fibrosis secondary to microaspiration due to gastroesophageal reflux disease (GERD) have previously been published.^[10]^ Patients with various forms of bronchiolitis were included. All HRCT scans were read by experienced radiologists or pulmonologists who registered their findings in a standardized form. Only inspiratory scans were included. Features indicative of underlying pulmonary fibrosis on HRCT included honeycombing, traction bronchiectasis, lung architectural distortion, and reticulation.

Values for age and forced vital capacity (FVC)% predicted were expressed by mean and standard deviation and final diagnoses and their association with squawks by number and percentage. Comparisons between patients with and without squawks were made by Student *t* test for age and FVC% and by chi-square test for proportions. All statistical analyses were performed using Statistical Package for Social Science (SPSS, version 22, IBM, Armonk, NY).

The study protocol was approved by the Ethical Committee of the hospital.

## 3 Results

One thousand consecutive cases with ILD were included. In total, 57.7% were women. Mean age was 60.0 ± 14.1 years. The diagnoses were supported by surgical lung biopsy in 20.8%, transbronchial biopsy in 9.6%, and biopsy from other sites in 3.1% of cases.

Final diagnoses, in decreasing order of occurrence, were the following: connective tissue disease (CTD; 24.8%), HP (21.6%), IPF (9.7%), sarcoidosis (7.5%), fibrosis secondary to microaspiration (7.1%), familial interstitial lung disease (FILD; 4.0%), drug-induced lung diseases (3.4%), bronchiolitis (2.9%), cryptogenic organizing pneumonia (COP; 2.6%), and others (16.4%).

Squawks were recorded in 49 cases (4.9%). There was no statistical difference between the frequency of squawks auscultated by the main investigator and research fellows (4.8% and 5.0%, respectively, *P* = .88). General and HRCT findings in patients with and without squawks are compared in Table 1. Patients with squawks had lower values of FVC% and a higher percentage of mosaic pattern and findings indicative of fibrosis on HRCT. Environmental exposure for possible HP was common, and did not differ in patients with and without squawks.

**Table 1:**
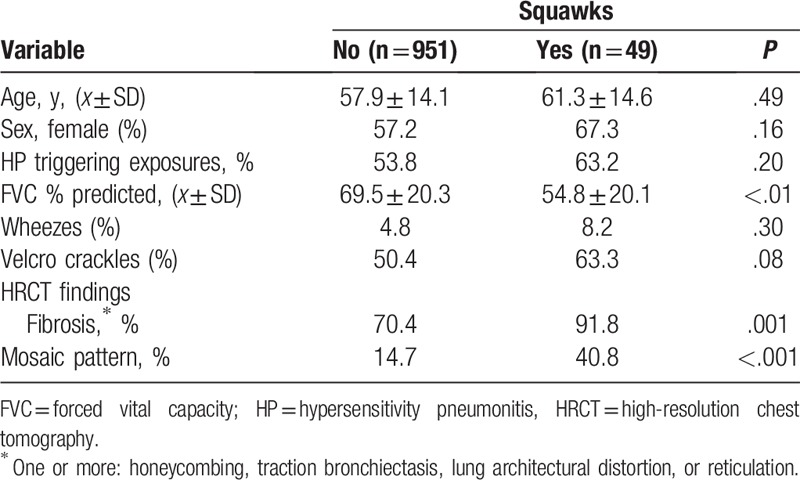
General and high-resolution computed tomography findings in patients with interstitial lung diseases, separated according presence or absence of squawks.

Wheezes were described in 50 cases, but there was no association between wheezes and squawks: Four (8.2%) with wheezes had squawks compared with 46 (4.8%) without squawks (*x*^2^ = 1.08, *P* = .30). Similarly, there was no significant association between squawks and Velcro crackles: in 510 cases with Velcro crackles, 31 (6.1%) had squawks compared with 18 (3.7%) of those 490 without Velcro crackles (*x*^2^ = 3.10, *P* = .08).

On HRCT, a mosaic pattern was described in 160 cases, being the sole finding in 12. The most common causes in patients with mosaic pattern were the following: HP (n = 86, 53.8%), bronchiolitis (n = 18, 11.2%), microaspiration due to GERD (n = 18, 11.2%), CTD (n = 12, 7.5%), sarcoidosis (n = 6, 3.8%), FILD (n = 2. 0.9%), and others (n = 18, 11.2%). There was a significant association between mosaic pattern and squawks: 20 cases with squawks (40.8%) had mosaic compared with 140 (14.7%) of those without squawks (*x*^2^ = 23.6, *P* < .001). Findings indicative of fibrosis were described on HRCT in 715 cases (71.5%). Squawks were more common in patients with findings indicative of fibrosis on HRCT: 45 of 715 (6.3%) compared with 4 of 285 (1.4%) of those without findings indicative of fibrosis (*x*^2^ = 10.46, *P* = .001).

Squawks were heard in decreasing order in HP (23 cases), CTD (7), microaspiration (4), pleuroparenchymal fibroelastosis (PPFE; 4), fibrosing COP (3), FILD (2), sarcoidosis (2), IPF (1), constrictive bronchiolitis due to rheumatoid arthritis or HP (1) (exposure to molds, not submitted to biopsy), bronchiolitis secondary to chronic lung allograft dysfunction due to bone marrow transplantation (1), and nonspecific interstitial pneumonia (1). One 74-year-old man with squawks had a final diagnosis of IPF. The diagnosis was made by typical findings on HRCT in the absence of an apparent cause. Diagnoses of interstitial lung diseases separated according to the presence or absence of squawks are shown in Table 2.

**Table 2:**
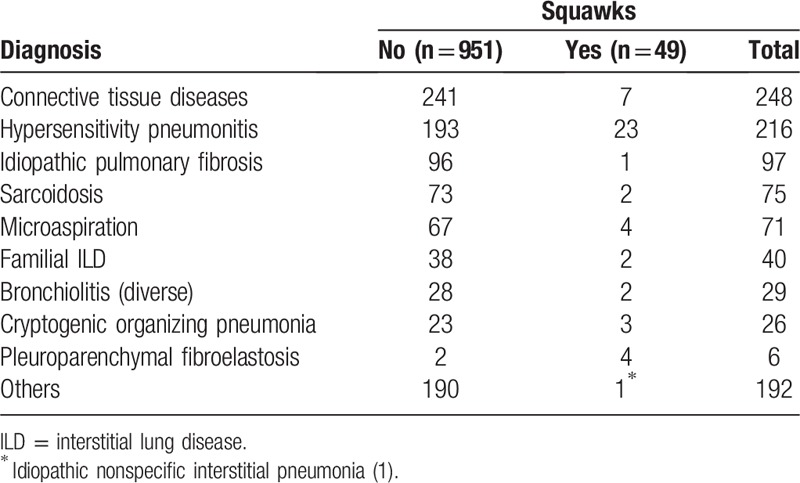
Diagnosis of interstitial lung diseases separated according presence or absence of squawks.

## 4 Discussion

Squawks are uncommon abnormal lung sounds, but the presence of these adventitious sounds in ILD narrows the differential diagnosis, pointing toward a group of fibrosing ILD with bronchiolar involvement. Some of these diseases are common, like HP and others are uncommon, like PPFE, in which squawks were not described before. Hypersensitivity pneumonitis is the most common cause. In IPF squawks are rare (1% of all cases of IPF in present series), confirming the data observed by Earis et al.^[8]^ Squawks are not exclusive to ILD. These sounds can be auscultated in bronchiectasis and pneumonias, due to bronchiolar involvement, but these are easily differentiated from ILD.^[3,18]^ One case with IPF and pneumonia with squawks was excluded from the present study.

In his classic monograph on lung sounds, Forgacs^[19]^ described inspiratory musical sounds in patients with various forms of diffuse interstitial pulmonary fibrosis. He postulated that these sounds were produced by sudden airways opening late in inspiration in areas of the lung deflated due to ILD. In 1982, the term inspiratory “squawk” was coined for this finding by Earis et al.^[8]^ In their study, 14 patients with squawks were described, all with conditions associated with diffuse pulmonary fibrosis. In 9 cases, this was due to HP.

We found an association between findings indicative of fibrosis on HRCT and the presence of squawks. This can be explained by the greater elastic forces involved in oscillations of the small airways during inspiratory opening of small airways in fibrotic ILD. Several ILD can involve small airways. Centrilobular or airway-centered accentuation of fibrosis is an important diagnostic clue suggesting chronic HP.^[10]^

Squawks have been described in sarcoidosis, and peripheral airway involvement is well recognized in this disease.^[20,21]^

Of 7 cases with squawks and CTD, 3 had rheumatoid arthritis (all with ILD), 3 had systemic sclerosis with GERD, and 1 had antisynthetase syndrome and a history of antigen exposure. Involvement of the small airways, whether isolated or associated with ILD or bronchiectasis, is common in rheumatoid arthritis.^[22]^ Idiopathic bronchiolocentric fibrosis (BCF) is a rare form of interstitial pneumonia. BCF secondary to microaspiration can be primary or secondary to GERD due to systemic sclerosis or, more commonly, it may be a pathologic expression of HP.^[10,23]^

In the present series, all 3 cases with COP were fibrosing, a variant now well recognized.^[24]^ Squawks in FILD, an increasingly recognized entity, were observed in 2 cases. This can occur due to airway-centered scarring or bronchiolitis. Mosaic pattern was seen on HRCT in these 2 cases, a finding described by others.^[25]^

In the present series, 6 cases had PPFE, and squawks were present in 4. We found no reports in the literature of squawks in this condition. PPFE classically has a subpleural distribution, but peribronchiolar extension of fibroelastosis is commonly found on pathological samples, and sometimes it is the predominant finding.^[9,26]^ In PPFE, an elevated residual volume/total lung capacity ratio is also commonly described in cases with air trapping on HRCT.^[27]^

The main strength of the present study was the inclusion of a large sample size of consecutive ILD, all submitted to lung auscultation in initial clinical evaluation by trained observers.

Nevertheless, some limitations of the study need to be considered. The concordance between observers for the presence of squawks was not tested, but the diseases associated with squawks, diagnosed in most cases after the initial evaluation, commonly involve small airways. In the last few years, diverse set of criteria was proposed for the diagnosis of HP.^[17,28,29]^ In the present study, criteria suggested by Salisbury et al were selected and retrospectively applied to all cases, but the final diagnosis of HP could change if other criteria were selected.

In the present study, mosaic patterns were observed in 16.0% of the sample, more than squawks, observed in 4.9% of cases. There was a significant association between the presence of squawks and mosaic pattern, but in 59% of cases in which squawks were found, mosaic patterns on inspiratory HRCT were not observed. HRCT scans in expiration were not performed in many of the patients and air trapping could be present. Some ILDs result in airflow obstruction, but the influence of this finding on the presence of squawks was not examined.^[30]^ Parenchymatous fibrosis, by increasing elastic recoil, can make airflow limitation due to bronchiolar involvement in apparent in spirometry.^[31]^

Findings indicative of fibrosis on HRCT were associated with the presence of squawks, but the extension of fibrosis in individual cases was not estimated.

Patients with squawks had, however, significant lower values of FVC. HP is the second most common cause of ILD in our center. However, the association of potential exposure with squawks for diagnosis of HP in Brazil is limited due to high prevalence of exposure to one or more potential causing agents for HP, mainly molds and birds at home.^[1]^

In summary, squawks are an uncommon finding on physical examination in patients with fibrosing ILD, but when present suggest ILDs associated with bronchiolar involvement. Squawks are rare in IPF.

## Author contributions

**Conceptualization:** Carlos A.C. Pereira.

**Formal analysis:** Carlos A.C. Pereira.

**Investigation:** Carlos A.C. Pereira, Maria R. Soares, Rafaela Boaventura, Marina D.C. Castro, Paula S. Gomes, Andrea Gimenez, Cesar Fukuda, Milena Cerezoli.

**Methodology:** Carlos A.C. Pereira.

**Writing – original draft:** Carlos A.C. Pereira, Maria R. Soares, Rafaela Boaventura, Marina D.C. Castro, Paula S. Gomes, Andrea Gimenez, Cesar Fukuda, Milena Cerezoli.

**Writing – review and editing:** Carlos A.C. Pereira, Maria R. Soares, Rafaela Boaventura, Marina D.C. Castro, Paula S. Gomes, Andrea Gimenez, Cesar Fukuda, Milena Cerezoli.

Carlos A.C. Pereira orcid: 0000-0002-0352-9589.

Carlos Pereira orcid: 0000-0002-0352-9589.
